# Factors Contributing to Pneumococcal, COVID-19, and Influenza Vaccine Uptake Among People Living With HIV in Belgium: A Retrospective Study

**DOI:** 10.1093/ofid/ofaf513

**Published:** 2025-09-03

**Authors:** Li-Cécile Destordeur, Victoria Lopez Delhoulle, Iraklis Papadopoulos, Nathalie Maes, Karine Fombellida, Majdouline El Moussaoui, Gilles Darcis

**Affiliations:** University of Liège, Liège, Belgium; University of Liège, Liège, Belgium; Biostatistics and Research Method Center, University Hospital of Liège, Liège, Belgium; Biostatistics and Research Method Center, University Hospital of Liège, Liège, Belgium; Infectious Diseases Department, Liège University Hospital of Liège, Liège, Belgium; Internal General Medicine, Liège University Hospital of Liège, Liège, Belgium; Infectious Diseases Department, Liège University Hospital of Liège, Liège, Belgium

**Keywords:** HIV, vaccination coverage, vaccine, vaccine hesitancy

## Abstract

**Background:**

Despite antiretroviral therapy, people living with HIV (PLWH) remain vulnerable to vaccine-preventable diseases. Although vaccination is strongly recommended, data on vaccine uptake among PLWH in Belgium remain scarce. This study aims to assess pneumococcal, COVID-19, and influenza vaccine coverage in PLWH in Belgium and identify factors associated with vaccine uptake.

**Methods:**

We conducted a retrospective study using the data from the HIV database of the Liege University Hospital in Belgium from 2017 to 2022. We evaluated vaccine coverage and collected demographic, clinical, and biological data to assess factors associated with vaccine uptake. Vaccine adherence was characterized as follows: partial adherence: receipt of at least one of the recommended vaccines during the study period and complete vaccination: pneumococcal vaccination, full COVID-19 primary vaccination, and annual influenza vaccination throughout the study period.

**Results:**

Among 791 participants, 89.1% received at least 1 dose of COVID-19 vaccine. Sixty-eight percent received at least 1 dose of influenza vaccine, but only 10.1% were vaccinated annually. Pneumococcal vaccine coverage was only 37.8%. Complete vaccine adherence was correlated with age (adjusted odds ratio [aOR]: 1.02, *P* = .024). Partial vaccine adherence was associated with age (aOR: 2.66, *P* = .026) and number of consultations (aOR: 1.23, *P* = .0002) and negatively associated with intravenous drug use (aOR: 0.15, *P* = .015).

**Conclusions:**

While COVID-19 vaccine uptake was high, vaccination coverage for influenza and pneumococcal disease remains insufficient. Age, healthcare encounters, and drug use were key factors influencing vaccine uptake. Targeted interventions and vaccine reminders should be conducted to increase vaccination rates.

## Teaser

This study investigates vaccine coverage among people with HIV in Belgium, revealing high COVID-19 vaccine uptake but insufficient influenza and pneumococcal vaccination rates. Factors such as age and intravenous drug use affect vaccine adherence, highlighting the need for targeted interventions.

Vaccination is an effective method to reduce complications and mortality related to vaccine-preventable diseases, especially in immunocompromised individuals. Despite advances in antiretroviral therapies (ART), people with HIV (PWH) remain more susceptible to preventable diseases compared with the general population [1, 2]. Given these risks, it is imperative to ensure optimal vaccine uptake among this population. Despite immune responses to vaccines being less effective in PWH [3, 4], vaccination is strongly recommended for them, preferably after having achieved suppressed viremia and immune reconstitution [5]. Nevertheless, vaccination rates have been reported to be below recommendations among PWH [6, 7]. For example, a study from Greece reports 39% influenza vaccination coverage among PWH, which highlights a gap in adherence [8].

Multiple factors contribute to lower vaccination rates in PWH, such as lower education level or lack of insurance coverage [9, 10]. Additionally, vaccine hesitancy, driven by a lack of knowledge about the benefits of vaccines, fear of potential side effects, and misconceptions about vaccine safety and efficacy, further reduces vaccination rates in this population and globally [11–14].

In Belgium, the Superior Health Council (SHC) recommends pneumococcal vaccination for adults at increased risk of pneumococcal disease [15, 16]. However, reimbursement is only partial and limited to individuals between 65 and 80 years old, which may impact uptake. Four types of pneumococcal vaccines are currently available: the PPV23 (pneumococcal polysaccharide vaccine), PCV13 (13 valent pneumococcal conjugate vaccine), PCV15 (15 valent pneumococcal conjugate vaccine), and PCV20 (20 valent pneumococcal conjugate vaccine). In 2020, the SHC recommended immunizing patients at risk of pneumococcal infection with 1 dose of PCV13, followed by 1 dose of PPV23 8 weeks later [17]. In 2022, the guidelines changed and the SHC recommended 1 administration of PCV20 followed by 1 dose of PPV23 5 years later [18].

The SHC also recommends annual influenza vaccination for immunocompromised individuals [19]. The cost of the vaccine is partially reimbursed by the INAMI (National Institute for Health and Disability Insurance) for high-risk patients [20].

Regarding SARS-CoV-2 vaccine in Belgium, the national vaccination campaign began in December of 2020 in nursing homes and rapidly expanded to the general population [21]. By October of 2021, vaccination rates had reached 86.4% of the population. The National public health institute of Belgium recommended an additional dose of COVID-19 vaccine for immunocompromised patients. COVID-19 vaccination was publicly funded, and the vaccines were free of charge.

There is limited literature about factors associated with adherence to vaccination among HIV infected people in Europe. This study aims to investigate pneumococcal, influenza, and COVID19 vaccination coverage in people living with HIV (PLWH) in Belgium as well as the factors influencing these rates. By identifying determinants of suboptimal vaccination uptake, we aim to support targeted strategies and informational campaigns to improve vaccine coverage in this vulnerable population.

## METHODS

This study is a single-center retrospective observational study conducted at the infectious disease clinic of the Liege University Medical Centre in Belgium. Data were extracted from center's HIV database, spanning 2017–2022. Physicians entered data into the database during routine outpatient consultations. Electronic health records were consulted to fill in gaps in data.

This study included individuals with HIV who were aged 18 years and older and who received care at the Liege University Medical Centre's infectious disease clinic. Individuals who had <2 years of follow-up were excluded from the study.

We assessed the adherence to influenza, COVID-19, and pneumococcal (PCV13, PPV23, PCV15, and PCV20) vaccines. Adherence to pneumococcal vaccination was defined as having received at least 1 dose of any pneumococcal vaccine (PCV13, PPV23, and PCV20) during the study period. Influenza vaccine uptake was assessed every vaccination season (from September to February). A participant was deemed adherent if they had received the vaccine during the season. Participants were deemed adherent to COVID-19 vaccination if they had received at least 1 dose of any COVID-19 vaccine.

We collected demographic, clinical, and biological data such as age (categorized as <50 and >50), sex, BMI, ethnicity, alcohol consumption, smoking status, and number of consultations per year. We also documented HIV-related data, namely nadir CD4 cell count and mode of transmission (male-to-male sexual contact, intravenous drug use, heterosexual sexual contact, mother-to-child transmission). Employment status was also recorded and categorized as employed, unemployed, or retired.

### Statistical Analysis

Continuous variables were described using means and standard deviations, medians and interquartile ranges (Q1–Q3), and extreme values (minimum, maximum). Qualitative variables were described using frequency tables (numbers and percentages).

To study the associations between vaccine adherence and the characteristics of PWH, univariate and multivariate logistic regression analyses were applied to 5 different adherence outcomes:

- Partial adherence: receipt of at least 1 dose pneumococcal vaccine and/or at least 1 primary COVID-19 vaccination and/or at least 1 influenza vaccination.
- Complete adherence: receipt of at least 1 dose of pneumococcal vaccine, at least 1 primary COVID-19 vaccination, and an annual influenza vaccination during the study period.
- Individual adherence to pneumococcal, COVID-19, and influenza vaccinations.

Odds ratios (OR) with 95% confidence intervals are presented for each variable and estimated for the complete case data, along with *P*-values.

Confounder control was implemented through multivariate logistic regression models. Variables included in the multivariate model were selected based on associations observed in univariate logistic regression.

The analyses were conducted using R software (version 4.3.2). The analyses were performed on the maximum available data; missing values were not imputed. The results were considered significant at the 5% threshold (*P* < .05).

### Ethical Considerations

Approval for the study protocol was obtained from the local ethics review committee (Comité d’Ethique Hospitalo-Facultaire Universitaire de Liège), reference number 2023-260. Participants were informed of data collection by their treating physician and could object to further collection of clinical data. All participants included were assigned unique identification numbers to anonymize the data and protect confidentiality. All methods were carried out in accordance with relevant guidelines and regulations.

## RESULTS

A total of 791 participants met the inclusion criteria and were included in our study (Supplementary Table 1). Of them, 364 (46%) were women. The median age was 45.2 years. 51.6% of participants were of African origin and 44.6% were Caucasian (Supplementary Table 1). The main mode of contamination was heterosexual contact (59.8%), followed by male-to-male sexual contact (30.5%). Twelve participants acquired HIV through IV drug use and 14 through mother-to-child transmission. The median nadir CD4 was 256. Sixty-six percent of participants reported alcohol consumption, while 64% were not smokers. Forty-one participants reported drug use. The median BMI was 25.8 (kg/m^2^). Four hundred and sixty-eight participants were employed, 45 were retired, and 85 were unemployed (Supplementary Table 1).

### Pneumococcal Vaccination

A total of 299 participants (37.8%) received at least 1 dose of a pneumococcal vaccine (Table 1). Of them, 142 received both the PCV13 and the PPV23, 96 participants only received the PPV23, 55 only received the PCV13, and 3 received the PCV20. No participants had received the PCV15 vaccine. Two individuals received a combination of PPV23, PCV13 and PCV20, which is not recommended.

**Table 1.** Overall View of Pneumococcal, Influenza, and COVID-19 Vaccinations Between 2017 and 2022 (N = 791)

Variables	Categories	N (%)
Pneumococcal vaccination	None	492 (62.2)
	At least once	299 (37.8)
	Only PCV20	3 (0.4)
	Only PPV23	96 (12.1)
	Only PCV13	55 (7.0)
	PCV20 + PPV23	1 (0.1)
	PCV20 + PCV13	0 (0.0)
	PPV23 + PCV13	142 (18.0)
	PCV20 + PCV13 + PPV23	2 (0.3)
	PCV15	0 (0.0)
Influenza vaccination	None	173 (21.87)
	At least once	618 (78.1)
	Every year	80 (10.1)
COVID-19 vaccination	None	86 (10.9)
	1 dose	705 (89.1)
	2 doses	674 (85.2)
	3 doses	497 (62.8)

**Figure 1.**
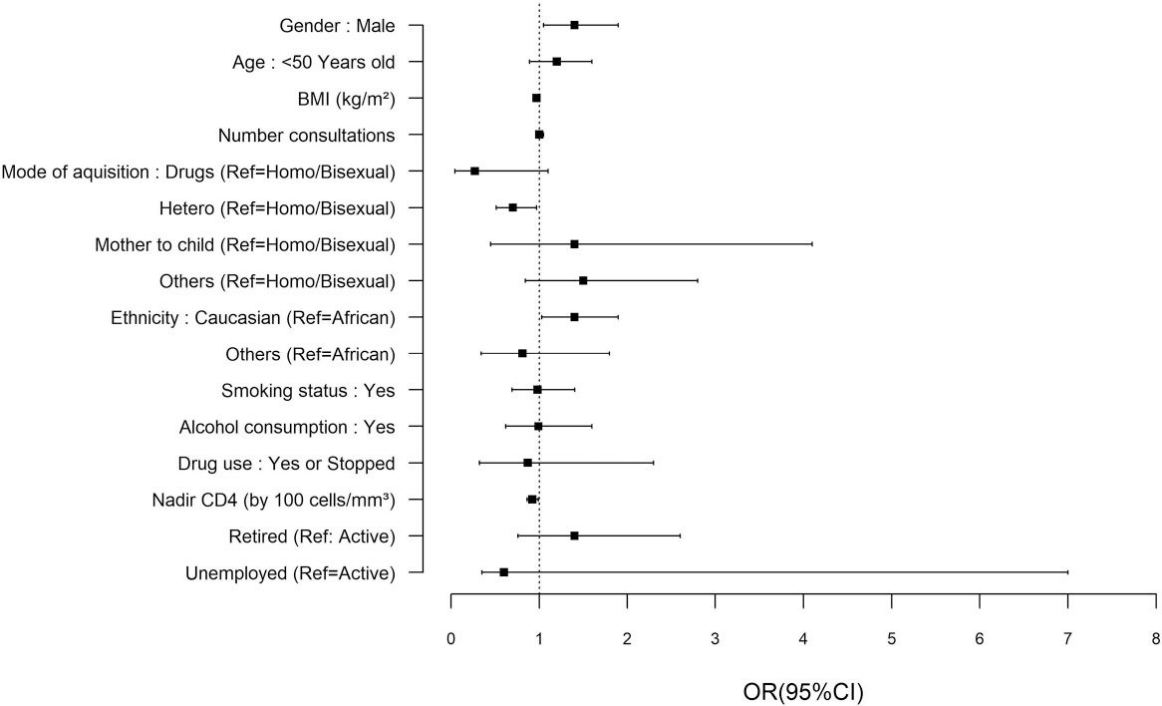
Factors associated with pneumococcal vaccine adherence (odd ratios and 95% confidence interval from univariate logistic regression).

Pneumococcal vaccine uptake was associated with age (OR 1.01, *P* = .02) and male gender (OR 1.4, *P* = .022) (Figure 1 and Supplementary Table 2). It was inversely associated with heterosexual transmission (OR 0.70, *P* = .03), nadir CD4 (OR 0.99, *P* = .034), and Caucasian ethnicity (OR 1.4, *P* = .03) (Figure 1). However, the associations with gender, ethnicity, and mode of contamination were not maintained after we adjusted for confounding factors. BMI, number of consultations, employment status, smoking, and alcohol consumption did were not associated with pneumococcal vaccine adherence (Figure 1).

### Influenza Vaccination

**Figure 2.**
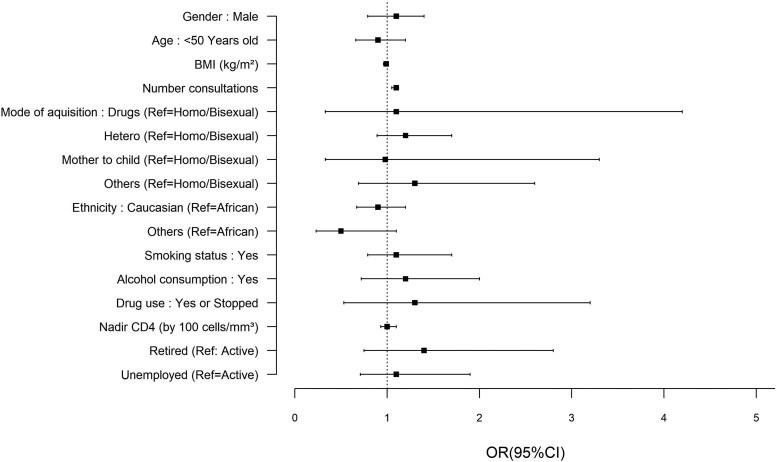
Factors associated with Influenza vaccine adherence (odd ratios and 95% confidence interval from univariate logistic regression).

Sixty-eight percent of participants were vaccinated against influenza at least once during the study period (Table 1). However, only 80 participants were vaccinated every season from 2017 to 2022 (Table 1). Influenza vaccine uptake was higher among participants who had more frequent consultations (OR 1.1, *P* < .0001) (Figure 2 and Supplementary Table 3). No significant associations were observed with smoking status, BMI, employment status, and age (Figure 2).

### COVID-19 Vaccination

COVID-19 vaccine uptake was the highest among the 3 vaccine types. During the study period, 89.1% of participants received at least 1 dose, 85.2% received 2 doses, and 62.8% received 3 doses (Table 1).

**Figure 3.**
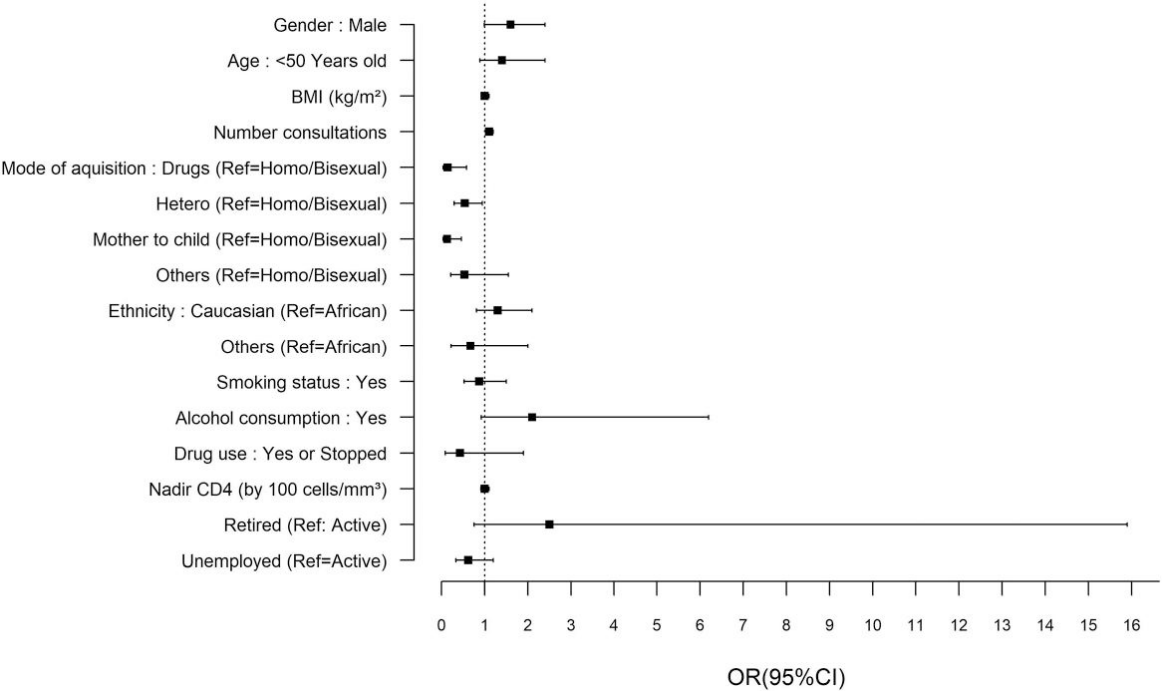
Factors associated with COVID-19 vaccine adherence (odd ratios and 95% Confidence interval from univariate logistic regression).

Adherence to COVID-19 vaccination was positively associated with number of consultations (aOR 1.08, *P* = .014), indicating that each additional consultation increased the odds of vaccination. COVID-19 vaccine uptake was inversely associated with IV drug use as the reported mode of HIV transmission (aOR 0.15, *P* = .005), heterosexual contact (aOR 0.55, *P* = .045), and mother-to-child transmission (aOR 0.12, *P* < .001) (Figure 3 and Supplementary Table 4). However, self-reported drug use itself regardless of transmission route was not independently associated with vaccine uptake.

### Partial and Complete Vaccine Adherence

Seven hundred and fifty-five participants received at least 1 dose of any of the vaccines we assessed. In terms of partial adherence (Supplementary Table 5), participants with more frequent consultations were more likely to be partially vaccinated (aOR 1.23, *P* = .0002). Age was also correlated with partial adherence (aOR 2.66, *P* = .026). Partial adherence was inversely associated with IV drug use as a mode of contamination (aOR 0.15, *P* = .015). No associations were found with smoking status, employment status, BMI, or alcohol consumption (Supplementary Table 5).

Regarding complete vaccine uptake, only age (aOR 1.02, *P* = .024) and nadir CD4 (OR 0.99, *P* = .037) were significantly associated (Supplementary Table 6). Smoking status, BMI, employment status, mode of contamination, and alcohol consumption did not significantly impact complete vaccine uptake.

## DISCUSSION

To our knowledge, this is the first study about vaccine coverage for respiratory infections among PWH in Belgium. Our findings reveal significant disparities in adherence across vaccine types and demographic groups, with overall suboptimal vaccination coverage.

Pneumococcal vaccine uptake in our cohort was 37.8%, which reflects suboptimal adherence to vaccination guidelines. Another study from Greece reported a much higher vaccination rate of 79%, potentially explained by the fact that the vaccines were free of charge [22]. However, even with free vaccines, the Greek study still identified lack of insurance coverage and low education as factors associated with lower pneumococcal vaccine uptake, highlighting the importance of educational campaigns to raise awareness among at-risk individuals.

In Belgium, pneumococcal vaccination is not fully reimbursed by the INAMI (National Institute for Health and Disability Insurance). The cost ranges from 3 366€ for the PPV23 to 75.55€ for the PCV20 and the PCV13 [27]. In our study, 96 participants only received the PPV23, which is the least expensive pneumococcal vaccine. This may reflect financial constraints in some of our participants. However, in our cohort, employment status was not significantly associated with vaccination rates. Unfortunately, the lack of comprehensive socioeconomic data beyond employment status limits our ability to fully assess the impact of financial barriers. Other studies reported vaccination rates ranging from 4.4% to 94.5% [8, 23–25]. Those variations might be explained by differences in study design, population, or healthcare infrastructure. A Belgian study reported similar pneumococcal vaccination rate of 32% among other at-risk group, identifying a lack of information about the pneumococcal vaccine as a major barrier [26]. In a Danish study reporting a vaccination rate of 4.4%, only 6.4% of participants were offered vaccination by their healthcare provider [8]. These findings underscore the impact of insufficient information and guidance regarding vaccination on immunization rates.

Enhancing healthcare provider education about vaccination guidelines could improve physicians’ awareness about vaccination and might increase their likelihood of offering vaccines, ultimately contributing to higher immunization rates. In our center, pneumococcal vaccination is typically offered during HIV follow-up visits by the infectious disease specialists; however, vaccines are not available on site. Patients usually get vaccinated by their general practitioner. The lack of on-site vaccination could possibly contribute to lower vaccine uptake, as it introduces additional logistical steps. These include the need to schedule separate appointments, potential transportation issues, or the risk that the patient may deprioritize or forget the recommendation altogether, ultimately acting as barriers to timely vaccination.

Older age was positively correlated with higher vaccine adherence in our study, possibly reflecting increased awareness of the risks associated with pneumococcal infections or more frequent healthcare interactions among older participants.

Influenza vaccination uptake was higher, with 68% of participants receiving at least 1 dose during the study period. This is encouraging but still highlights a gap, as only a small fraction (10.1%) of participants was vaccinated consistently across all seasons from 2017 to 2022. Frequent healthcare consultations were a significantly associated with influenza vaccination, suggesting that regular contact with healthcare providers enhances opportunities for vaccination. This aligns with previous research indicating that routine clinical visits provide critical opportunities for participants’ education and vaccination [24].

Smoking status did not influence influenza vaccine uptake, despite the known association between smoking and severe influenza outcomes, such as hospitalizations and ICU admission [28, 29]. This indicates a need for targeted interventions to increase vaccine uptake among smokers. A Belgian study showcased an influenza vaccination rate of 44% among other at-risk groups, which is slightly lower than in our cohort. Concerns about effectiveness and side effects were reported as barriers to uptake [26]. Similar findings were reported in studies from Denmark and Italy, where lack of information, perceived good health, and concern about side effects reduce uptake and where many participants had not been informed by their healthcare provider [8, 9]. These results reinforce the importance of improving the provider–patient communication around vaccines.

In Belgium, influenza vaccination can be offered by the infectious disease specialist during HIV follow-up consultations or, alternatively by the general practitioner. The vaccines are often available on site at the AIDS resource centre, and the patients can receive their vaccinations during their clinic visit, an approach that may improve vaccine uptake.

COVID-19 vaccine uptake was the highest among the vaccinations we studied, with 89.1% of participants receiving at least 1 dose. This is comparable to national rates, reported by Sciensano, the National Public Health Institute of Belgium [30]. This high rate of vaccination likely reflects the intensive public health efforts and prioritization of COVID-19 vaccination during the pandemic, along with the increased perceived risk of COVID-19, particularly in immunocompromised populations. The COVID-19 pandemic represented a unique context, during which vaccination became quasimandatory due to widespread restrictions. In October 2021, Belgium introduced the COVID Safe Ticket pass. People were required to show proof of full COVID-19 vaccination or a negative polymerase chain reaction test to access various public and private facilities [31].

However, this exceptional context may have artificially elevated COVID-19 vaccine uptake compared to other vaccines, limiting its value as a direct comparator of adherence behavior. Moreover, the COVID-19 vaccination campaign was publicly funded, and the vaccines were free of charge and widely available at local vaccination centers. These findings suggest that reducing financial and logistical burden could improve vaccine adherence.

Individuals with more frequent healthcare interactions were more likely to be vaccinated. This trend could be attributed to several factors: regular consultations might provide physicians with more opportunities to encourage vaccination or record patients’ vaccination statuses. Additionally, individuals with more outpatient encounters might be more health-conscious and exhibit a more positive attitude toward vaccination. In our study, COVID-19 was not associated with age, which is inconsistent with another study that highlighted age as a factor for vaccine hesitancy [32].

The low vaccine uptake among participants who contracted HIV through IV drug use highlights the ongoing challenges in reaching certain at-risk populations and the need for tailored interventions that address specific barriers in this group. A Canadian study reported that lack of knowledge about vaccination was a frequent contributor to vaccine hesitancy among people who inject drugs [35]. Offering vaccinations at syringe distribution services was shown to be an efficient solution to reach drug users and promote vaccination to this vulnerable population [36].

However, this subgroup represented only a small percentage of the overall cohort and that there was substantial amount of missing data, which limits the strength of the conclusions that can be drawn.

When we take partial vaccine adherence into account, our study demonstrates that vaccine adherence is positively correlated with number of visits, which is consistent with other studies [33, 34]. Older participants were also more likely to be vaccinated.

Regarding complete adherence, adherence was higher among older participants and was inversely associated with nadir CD4. Other studies also showcased a higher vaccination rate in older individuals [8, 22]. In a study conducted at the Henry ford Hospital infectious disease clinic, vaccine adherence was associated with lower age, which is inconsistent with our findings. Our overall vaccination rates were much lower than those at the Henry Ford Hospital [23]. That clinic benefited from a philanthropic grant program that covers the cost vaccination, and implemented several vaccine reminder systems, 2 strategies which are proven to improve vaccine uptake [33, 37]. Implementing similar systems, such as a national vaccination registry system and automated vaccine reminders, might be an efficient way to improve vaccine coverage in our population. Furthermore, vaccine uptake may be improved by increasing vaccine reimbursement for PLWH.

### Limitations

Our study has some limitations. First, the study's retrospective design presents inherent limitations, particularly related to data completeness and accuracy. Because data were extracted from medical records, information such as vaccination obtained outside the hospital's setting has been missing or incomplete. Additionally, the reliance on routinely collected clinical data limits control over variable standardization and may introduce bias. We did not collect data about vaccine refusal, which limits our understanding of factors associated with vaccine hesitancy. Another limitation is the way we defined adherence as “having received at least one dose of a vaccine.” This may have slightly overestimated the true adherence rates. We also lacked data about the type of COVID-19 vaccine the participant had received, which prevented us from accurately evaluating whether participants had received a complete vaccine series. Another limitation of this study is the lack of comprehensive socioeconomic data beyond employment status. Important factors such as education level, income, and access to social support were not recorded in the medical records and therefore could not be included in the analysis. These factors are known to influence healthcare access and health literacy. Further research should address these variables to better understand the social determinants to vaccine uptake.

Despite these limitations, our study's large sample size is a significant strength, providing robust data that can inform public health strategies. Future research should aim to collect prospective data, include information on vaccine refusal, and consider the completion of vaccination series to provide a more comprehensive picture of vaccine adherence.

## CONCLUSION

Our study provides valuable insights into vaccine coverage for respiratory infections among PLWH in Belgium, highlighting significant disparities in vaccine adherence across demographic, clinical, and socioeconomic factors. While COVID-19 vaccine uptake was notably high, likely due to public health initiatives, free vaccine access, and pandemic-related policies, adherence to pneumococcal and influenza vaccination guidelines remains suboptimal. Key predictors of higher vaccination rates included older age and more frequent healthcare interactions, emphasizing the critical role of regular medical contact in promoting vaccine adherence. Improving general practitioners’ knowledge about vaccination guidelines may also increase opportunities to offer vaccination to this at-risk group.

The findings underscore the persistent challenges in reaching marginalized populations, such as intravenous drug users, and the need for tailored interventions, including integrating vaccinations into harm reduction programs. Additionally, addressing financial barriers, improving education about the benefits of vaccination, and implementing reminder systems could significantly enhance vaccine uptake in this vulnerable population. Implementing on-site vaccination after routine HIV follow-up visits may also improve vaccine uptake.

Future efforts should focus on prospective data collection, including vaccine refusal factors and completion of vaccination series, to better understand and address barriers to adherence. By adopting multifaceted strategies, public health initiatives can improve vaccination rates, reduce health disparities, and better protect PLWH from preventable respiratory infections.

## Supplementary Material

ofaf513_Supplementary_Data

## Supplementary Data

Supplementary materials are available at *Open Forum Infectious Diseases* online. Consisting of data provided by the authors to benefit the reader, the posted materials are not copyedited and are the sole responsibility of the authors, so questions or comments should be addressed to the corresponding author.
