# Supplementary material for: Factors Contributing to Pneumococcal, COVID-19, and Influenza Vaccine Uptake Among People Living With HIV in Belgium: A Retrospective Study
Source: Open Forum Infect Dis. 2025 Sep 3;12(9):ofaf513. doi: 10.1093/ofid/ofaf513 (PMC12405995; doi:10.1093/ofid/ofaf513)
Supplement: ofaf513_Supplementary_Data [file ofaf513_supplementary_data.docx]

**Supplementary :**

| **Supplementary 1: Characteristics of participants (N=791)** | | | |
| --- | --- | --- | --- |
|  | Category | N | Results |
| Gender | Female  Male | 791 | 364 (46.0%)  427 (54.0%) |
| Age on 1/01/2017 (years) | Mean (SD)  Median [Q1, Q3]  [Min, Max] | 791 | 46.0 (11.8)  45.2 [37.6, 54.9]  [19.0, 85.0] |
| Ethnicity | African  Caucasian  Other | 791 | 408 (51.6%)  353 (44.6%)  30 (3.8%) |
| Mode of contamination | Drugs  Heterosexual  Homosexual/Bisexual  Mother to child transmission  Other | 791 | 12 (1.5%)  473 (59.8%)  241 (30.5%)  14 (1.8%)  51 (6.4%) |
| Nadir CD4 (cells/mm^3^) | Mean (SD)  Median [Q1, Q3]  [Min, Max] | 791 | 287 (206)  256 [140, 396]  [0, 1310] |
| Alcohol consumption | Stopped <1 year  Stopped ≥1 an  Never  Yes | 687 | 9 (1.3%)  9 (1.3%)  211 (30.7%)  458 (66.7%) |
| Smoking status | Stopped<1 year  Stopped ≥1 year  Never  Yes | 766 | 22 (2.9%)  79 (10.3%)  490 (64.0%)  175 (22.8%) |
| Drug use | Never  Yes  Stopped <1 year  Stopped ≥1 year | 94 | 53 (56.4%)  33 (35.1%)  6 (6.4%)  2 (2.1%) |
| BMI (Kg/m²) | Mean (SD)  Median [Q1, Q3]  [Min, Max] | 790 | 26.4 (4.96)  25.8 [22.8, 29.4]  [15.0, 49.1] |
| Employment | Active  Retired  Unemployed | 598 | 468 (78.3%)  45 (7.5%)  85 (14.2%) |

| **Supplementary 2: Factors associated with pneumococcal vaccine adherence, univariate and multivariate logistic regression** | | | | | | | |
| --- | --- | --- | --- | --- | --- | --- | --- |
| **Variable** | **N** | **Categories** | **Reference** | **OR (95%CI)** | **p value** | **Adj. OR (95%CI) (N=460)** | **p value** |
| **Gender** | **791** | **Male** | **Female** | **1.4 (1.05, 1.9)** | **0.022** | **1.15 (0.78, 1.70)** | **0.466** |
| **Age (Years) on 01/01/2017** | **791** |  |  | **1.01 (1.002, 1.03)** | **0.020** | **1.01 (0.99, 1.02)** | **0.087** |
| **Age (< or ≥ 50 years old)** | **791** | **<50** | **≥50** | **1.2 (0.89, 1.6)** | **0.22** |  |  |
| **BMI (kg/m²)** | **790** |  |  | **0.97 (0.95, 1.0)** | **0.085** |  |  |
| **Number of consultations between 2017 and 2022** | **791** |  |  | **1.0 (0.97, 1.05)** | **0.58** |  |  |
| **Mode of Contamination** | **791** | **Drugs** | **Homosexual/Bisexual** | **0.27 (0.041, 1.1)** | **0.098** | **0.25 (0.04, 1.01)** | **0.085** |
|  |  | **Heterosexual** |  | **0.70 (0.51, 0.97)** | **0.030** | **0.79 (0.48, 1.30)** | **0.370** |
|  |  | **Mother-to-child** |  | **1.4 (0.45, 4.1)** | **0.57** | **1.90 (0.59, 6.07)** | **0.271** |
|  |  | **Others** |  | **1.5 (0.84, 2.8)** | **0.17** | **1.75 (0.89, 3.47)** | **0.101** |
| **Ethnicity** | **791** | **Caucasian** | **African** | **1.4 (1.03, 1.9)** | **0.030** | **1.20 (0.78, 1.83)** | **0.397** |
|  |  | **Other** |  | **0.81 (0.34, 1.8)** | **0.61** | **0.90 (0.37, 2.06)** | **0.824** |
| **Smoking status** | **766** | **Yes** | **Never/ stopped** | **0.98 (0.69, 1.4)** | **0.91** |  |  |
| **Alcohol consumption** | **687** | **Yes** | **Occasional/ Stopped ≥1 year/ Never** | **0.99 (0.62, 1.6)** | **0.97** |  |  |
| **Drug use** | **94** | **Yes or stopped** | **Never** | **0.87 (0.32 , 2.3)** | **0.77** |  |  |
| **Nadir CD4** | **791** | **(cells/mm^3^)** |  | **0.999 (0.998, 0.9999)** | **0.034** | **0.999 (0.998, 0.9999)** | **0.020** |
| **Employment** | **598** | **Retired** | **Active** | **1.4 (0.76 , 2.6)** | **0.27** |  |  |
|  |  | **Unemployed** |  | **0.60 (0.35 , 7.0)** | **0.051** |  |  |

| **Supplementary 3. Factors associated to Influenza vaccine adherence, univariate and multivariate logistic regression** | | | | | |
| --- | --- | --- | --- | --- | --- |
| **Variable** | **N** | **Categories** | **Reference** | **OR (95%CI)** | **p value** |
| **Gender** | **791** | **Male** | **Female** | **1.06 (0.79, 1.4)** | **0.69** |
| **Age (Years) on 01/01/2017** | **791** |  |  | **0.99 (0.98, 1.005)** | **0.22** |
| **Age (< or ≥ 50 years old)** | **791** | **<50** | **≥50** | **0.90 (0.66, 1.2)** | **0.50** |
| **BMI (kg/m²)** | **790** |  |  | **0.99 (0.96, 1.02)** | **0.59** |
| **Number of consultations between 2017 and 2022** | **791** |  |  | **1.1 (1.05, 1.1)** | **<0.001** |
| **Mode of Contamination** | **791** | **Drugs** | **Homosexual/Bisexual** | **1.1 (0.33, 4.2)** | **0.89** |
|  |  | **Heterosexual** |  | **1.2 (0.89, 1.7)** | **0.19** |
|  |  | **Mother-to-child** |  | **0.98 (0.33, 3.3)** | **0.97** |
|  |  | **Others** |  | **1.3 (0.69, 2.6)** | **0.42** |
| **Ethnicity** | **791** | **Caucasian** | **African** | **0.90 (0.67, 1.2)** | **0.52** |
|  |  | **Other** |  | **0.50 (0.23, 1.1)** | **0.068** |
| **Smoking status** | **766** | **Yes** | **Never/ stopped** | **1.1 (0.79, 1.7)** | **0.48** |
| **Alcohol consumption** | **687** | **Yes** | **Occasional/ Stopped ≥1 year/ Never** | **1.2 (0.72, 2.0)** | **0.51** |
| **Drug use** | **94** | **Yes or stopped** | **Never** | **1.3 (0.53, 3.2)** | **0.58** |
| **Nadir CD4** | **791** | **(cells/mm^3^)** |  | **1.0 (0.99, 1.0)** | **0.93** |
| **Employment** | **598** | **Retired** | **Active** | **1.4 (0.75, 2.8)** | **0.30** |
|  |  | **Unemployed** |  | **1.1 (0.71, 1.9)** | **0.57** |

| **Supplementary 4: Factors associated with COVID-19 vaccine uptake, univariate and multivariate analysis** | | | | | | | |
| --- | --- | --- | --- | --- | --- | --- | --- |
| **Variable** | **N** | **Categories** | **Reference** | **OR (95%CI)** | **p value** | **Adj. OR (95%CI) (N=791)** | **p value** |
| **Gender** | **791** | **Male** | **Female** | **1.6 (0.99, 2.4)** | **0.055** |  |  |
| **Age (Years) on 01/01/2017** | **791** |  |  | **1.0 (1.0, 1.0)** | **0.051** |  |  |
| **Age (< or ≥ 50 years old)** | **791** | **<50** | **≥50** | **1.4 (0.89, 2.4)** | **0.15** |  |  |
| **BMI (kg/m²)** | **790** |  |  | **1.0 (0.97, 1.1)** | **0.57** |  |  |
| **Number of consultations between 2017 and 2022** | **791** |  |  | **1.1 (1.02, 1.2)** | **0.013** | **1.08 (1.02, 1.17)** | **0.014** |
| **Mode of Contamination** | **791** | **Drugs** | **Homosexual/Bisexual** | **0.14 (0.040, 0.58)** | **0.003** | **0.15 (0.04, 0.61)** | **0.005** |
|  |  | **Heterosexual** |  | **0.54 (0.29, 0.94)** | **0.037** | **0.55 (0.29, 0.96)** | **0.045** |
|  |  | **Mother-to-child** |  | **0.13 (0.039, 0.46)** | **0.0008** | **0.12 (0.04, 0.43)** | **<0.001** |
|  |  | **Others** |  | **0.53 (0.21, 1.55)** | **0.21** | **0.58 (0.22, 1.68)** | **0.278** |
| **Ethnicity** | **791** | **Caucasian** | **African** | **1.3 (0.81, 2.1)** | **0.28** |  |  |
|  |  | **Other** |  | **0.67 (0.22, 2.0)** | **0.43** |  |  |
| **Smoking status** | **766** | **Yes** | **Never/ stopped** | **0.87 (0.52, 1.5)** | **0.62** |  |  |
| **Alcohol consumption** | **687** | **Yes** | **Occasional/ Stopped ≥1 year/ Never** | **2.1 (0.92, 6.2)** | **0.11** |  |  |
| **Drug use** | **94** | **Yes or stopped** | **Never** | **0.43 (0.084, 1.9)** | **0.27** |  |  |
| **Nadir CD4** | **791** | **(cells/mm^3^)** |  | **1.0 (0.999, 1.001)** | **0.73** |  |  |
| **Employment** | **598** | **Retired** | **Active** | **2.5 (0.75, 15.9)** | **0.21** |  |  |
|  |  | **Unemployed** |  | **0.62 (0.33, 1.2)** | **0.16** |  |  |

| **Supplementary 5: Factors associated with partial vaccine adherence (Pneumococcal or influenza or COVID-19), univariate and multivariate logistic regression.** | | | | | | | | |
| --- | --- | --- | --- | --- | --- | --- | --- | --- |
| **Variable** | **N** | **Categories** | **Reference** | **OR (95%CI)** | **p value** | **Adj. OR (95%CI) (N=791)** | **p value** |  |
| Gender | 791 | Male | Female | 1.1 (0.53 - 2.1) | 0.88 |  |  |  |
| Age (Years) on 01/01/2017 | 791 |  |  | 1.03 (1.004 - 1.07) | 0.031 |  |  |  |
| Age (< or ≥ 50 years old) | 791 | <50 | ≥50 | 2.6 (1.2 - 6.4) | 0.027 | 2.66 (1.19, 6.77) | 0.026 |  |
| BMI (kg/m²) | 790 |  |  | \| 1.0 (0.96,1.1) \| \| --- \| | 0.46 |  |  |  |
| Number of consultations between 2017 and 2022 | 791 |  |  | 1.2 (1.1 - 1.4) | 0.0004 | 1.23 (1.11, 1.38) | 0.0002 |  |
| Mode of Contamination | 791 | Drugs | Homosexual/Bisexual | 0.12 (0.028 - 0.59) | 0.004 | 0.15 (0.03, 0.79) | 0.015 |  |
|  |  | Heterosexual |  | 0.93 (0.39 - 2.0) | 0.85 | 0.98 (0.41, 2.17) | 0.961 |  |
|  |  | Mother-to-child |  | 0.23 (0.052 - 1.6) | 0.081 | 0.27 (0.06, 2.01) | 0.136 |  |
|  |  | Others |  | 0.62 (0.18 - 2.9) | 0.49 | 0.73 (0.20, 3.43) | 0.651 |  |
| Ethnicity | 791 | Caucasian | African | 1.3 (0.63 - 2.7) | 0.50 |  |  |  |
|  |  | Other |  | 0.32 (0.11 - 1.1) | 0.050 |  |  |  |
| Smoking status | 766 | Yes | Never/stopped | 1.19 (0.54, 3.01) | 0.682 |  |  |  |
| Alcohol consumption | 687 | Yes | Occasional/ Stopped ≥1 year/ Never | 4.6 (0.96, 81.7) | 0.14 |  |  |  |
| **Drug use** | **94** | **Yes or stopped** | **Never** | 0.77 (0.089, 6.6) | 0.79 |  |  |  |
| Nadir CD4 | 791 | (cells/mm^3^) |  | 1.0 (0.998 - 1.001) | 0.33 |  |  |  |
| **Employment** | **598** | **Retired** | **Active** | NA* | - |  |  |  |
|  |  | **Unemployed** |  | 0.42 (0.17, 1.1) | 0.062 |  |  |  |

| **Supplementary 6: Factors associated with complete vaccine adherence (Pneumococcal and yearly influenza and COVID-19), univariate and multivariate logistic regression.** | | | | | | | |
| --- | --- | --- | --- | --- | --- | --- | --- |
| **Variable** | **N** | **Categories** | **Reference** | **OR (95%CI)** | **p value** | **Adj. OR (95%CI) (N=791)** | **p value** |
| Gender | 791 | Male | Female | 1.3 (0.95, 1.7) | 0.11 |  |  |
| Age (Years) on 01/01/2017 | 791 |  |  | 1.02 (1.004, 1.03) | 0.010 | 1.02 (1.002, 1.028) | 0.024 |
| Age (< or ≥ 50 years old) | 791 | <50 | ≥50 | 1.2 (0.92, 1.7) | 0.15 |  |  |
| BMI (kg/m²) | 790 |  |  | 0.99 (0.96, 1.02) | 0.47 |  |  |
| Number of consultations between 2017 and 2022 | 791 |  |  | 1.0 (0.997, 1.08) | 0.070 |  |  |
| Mode of Contamination | 791 | Drugs | Homosexual/Bisexual | 0.37 (0.055, 1.4) | 0.20 |  |  |
|  |  | Heterosexual |  | 0.84 (0.61, 1.2) | 0.31 |  |  |
|  |  | Mother-to-child |  | 0.73 (0.20, 2.3) | 0.61 |  |  |
|  |  | Others |  | 1.6 (0.88, 3.0) | 0.12 |  |  |
| Ethnicity | 791 | Caucasian | African | 1.29 (0.96, 1.7) | 0.098 |  |  |
|  |  | Other |  | 0.67 (0.26, 1.5) | 0.37 |  |  |
| Smoking status | 766 | Yes | Never/stopped | 0.92 (0.64, 1.3) | 0.63 |  |  |
| Alcohol consumption | 687 | Yes | Occasional/ Stopped ≥1 year/ Never | 1.1 (0.68, 1.8) | 0.68 |  |  |
| Drug use | 94 | Yes or stopped | Never | 0.53 (0.17, 1.5) | 0.24 |  |  |
| Nadir CD4 | 791 | (cells/mm^3^) |  | 0.999 (0.998, 0.9999) | 0.037 | 0.999 (0.998, 0.9999) | 0.090 |
| Employment | 598 | Retired | Active | 1.4 (0.73, 2.6) | 0.31 |  |  |
|  |  | Unemployed |  | 0.67 (0.39,1.1) | 0.13 |  |  |
